# Association patterns and community structure among female bottlenose dolphins: environmental, genetic and cultural factors

**DOI:** 10.1007/s42991-022-00259-x

**Published:** 2022-11-02

**Authors:** Svenja M. Marfurt, Simon J. Allen, Manuela R. Bizzozzero, Erik P. Willems, Stephanie L. King, Richard C. Connor, Anna M. Kopps, Sonja Wild, Livia Gerber, Samuel Wittwer, Michael Krützen

**Affiliations:** 1 Evolutionary Genetics Group, Department of Anthropology, University of Zurich, 8057 Zurich, Switzerland; 2 School of Biological Sciences, University of Western Australia, Perth, WA 6009 Australia; 3 School of Biological Sciences, University of Bristol, Bristol, BS8 1TQ UK; 4 Department of Anthropology, University of Zurich, 8057 Zurich, Switzerland; 5 Biology Department, UMASS Dartmouth, North Dartmouth, MA USA; 6 Evolution and Ecology Research Centre, School of Biological, Earth and Environmental Sciences, University of New South Wales, Sydney, NSW 2052 Australia; 7 Cognitive and Cultural Ecology Research Group, Max Planck Institute of Animal Behaviour, Am Obstberg 1, 78315 Radolfzell, Germany; 8 Centre for the Advanced Study of Collective Behaviour, University of Konstanz, Universitätsstrasse 10, 78464 Constance, Germany

**Keywords:** Bottlenose dolphins, Culture, Homophily, Kinship, Matrilineal structure, Tool-use

## Abstract

Social structuring from assortative associations may affect individual fitness, as well as population-level processes. Gaining a broader understanding of social structure can improve our knowledge of social evolution and inform wildlife conservation. We investigated association patterns and community structure of female Indo-Pacific bottlenose dolphins (*Tursiops aduncus*) in Shark Bay, Western Australia, assessing the role of kinship, shared culturally transmitted foraging techniques, and habitat similarity based on water depth. Our results indicated that associations are influenced by a combination of uni- and biparental relatedness, cultural behaviour and habitat similarity, as these were positively correlated with a measure of dyadic association. These findings were matched in a community level analysis. Members of the same communities overwhelmingly shared the same habitat and foraging techniques, demonstrating a strong homophilic tendency. Both uni- and biparental relatedness between dyads were higher within than between communities. Our results illustrate that intraspecific variation in sociality in bottlenose dolphins is influenced by a complex combination of genetic, cultural, and environmental aspects.

**Supplementary Information:**

The online version contains supplementary material available at 10.1007/s42991-022-00259-x.

## Introduction

Group living is a common feature of many mammalian societies (Rubenstein and Wrangham 1986; Ward and Webster 2016). Benefits include reduced predation risk through better predator detection or dilution, improved access to food resources, as well as increased opportunity for social learning (Pulliam 1973; Alexander 1974; Foster and Treherne 1981; Van Schaik 1983; Coussi-Korbel and Fragaszy 1995). In addition, females benefit from assistance in offspring rearing and enhanced protection from male sexual coercion (Wrangham 1980; Smuts and Smuts 1993; Rubenstein 1994). Such benefits may outweigh the costs of resource competition and increased susceptibility to disease transmission (Alexander 1974; Rubenstein and Wrangham 1986; Lutermann et al. 2013).

An important parameter expected to correlate with sociality is the degree to which interacting individuals are related. The theory of kin selection (Hamilton 1964) posits that individuals should help relatives whenever inclusive fitness benefits outweigh the possible costs (Holekamp et al. 2006; Silk 2007; Frère et al. 2010; Best et al. 2013). There are numerous studies in female mammals demonstrating preferential association with relatives (Smith 2014). In African elephants (*Loxodonta africana*), for example, biparental relatedness predicted temporal group fission patterns where individuals remained with close relatives (Archie et al. 2006). Further, members of the same mitochondrial DNA (mtDNA) matrilines fused more readily than females from different matrilines (Archie et al. 2006). Other examples where increased associations between related females were detected include greater horseshoe bats (*Rhinolophus ferrumequinum*, Rossiter et al. 2002), sperm whales (*Physeter microcephalus*, Gero et al. 2008), short-beaked common dolphins (*Delphinus delphis*, Zanardo et al. 2018) and chimpanzees (*Pan troglodytes*, Foerster et al. 2015).

Despite the important role that relatedness plays in explaining female association patterns, benefits of group living can also be accrued by associating with unrelated females. At the most basic level, group formation based on by-product benefits such as the dilution effect (see Connor 1995) can favour associations among non-relatives. Unrelated females may form strong associations when it is advantageous to do so. Such strong associations in unrelated female feral horses (*Equus caballus*) increased both birth rates and survival and reduced the risk of male harassment (Cameron et al. 2009). In chimpanzees, strong social bonds, as measured via high pairwise affinity indices, were facilitated via sharing of similar ranging patterns rather than genetic relatedness (Langergraber et al. 2009).

Intrinsic traits shown to modify social organisation include socially learned, group specific behaviours, *i.e.*, animal culture (Laland and Hoppitt 2003; Laland and Galef 2009). For example, sympatric killer whale (*Orcinus orca*) ecotypes differing in their prey preferences showed distinct sociality (Baird 2000; Saulitis et al. 2000; Ford and Ellis 2013). The evolution of these divergent ecotypes appeared to be a consequence of stable cultural differences (Riesch et al. 2012). Similarly, sympatric Indo-Pacific bottlenose dolphin (*Tursiops aduncus*) communities differed considerably in their foraging specialisations, one of which was linked to cultural transmission of trawler-associated foraging (Chilvers and Corkeron 2001; Ansmann et al. 2012). After cessation of trawl-fisheries in the area, previous trawler-associating dolphins were no longer distinguishable from individuals that had never engaged in trawler-associated feeding techniques (Ansmann et al. 2012).

Extrinsic effects such as resource and habitat variability, in particular the spatial arrangement of both abiotic and biotic components, have been shown to influence intraspecific and intra-population variation in social behaviour (Louis et al. 2018; He et al. 2019). Habitat complexity influenced social connectivity and stability in sticklebacks (*Gasterosteus aculeatus*, Webster et al. 2013) and sleepy lizards (*Tiliqua rugosa*, Leu et al. 2016), suggesting that spatial arrangements of habitat components can influence social interactions and thus shape social networks and overall sociality (He et al. 2019).

The social organisation of bottlenose dolphins (*Tursiops truncatus*) in Doubtful Sound, New Zealand, correlated with their temporally and spatially variable fjord habitat, likely the result of ecological constraints in this population (Lusseau et al. 2003). Hawaiian spinner dolphins (*Stenella longirostris*) exhibited grouping patterns that appeared to be dependent upon the region and water depth of the archipelago in which they were observed (Andrews et al. 2010). Individuals formed stable, long-lasting groups with strong associations in the north-west, characterised by large deep-water stretches, but formed dynamic, continuously changing groups in the south-east of the archipelago, a mosaic pattern of suitable resting habitats with sheltered shallow waters (Andrews et al. 2010). In Shark Bay, Western Australia, the structure and behaviour of alliance forming male Indo-Pacific bottlenose dolphins varies systematically along a 50-km stretch of coastline that also exhibits marked variation in habitat type (Connor et al. 2017; Hamilton et al. 2019).

Apart from genetic and environmental correlates, the tendency of individuals to bond with similar others, termed ‘homophily’, has also been linked to social structure. In human societies, for instance, individuals with analogous characteristics like religion, nationality, age, or level of education were found to cluster (McPherson et al. 2001; Rivera et al. 2010; Newman 2018). Homophily has also been described in non-human animals (Fu et al. 2012). In rhesus macaques (*Macaca mulatta*), the analogous ‘principle of similarity’ was shown to determine attraction among females, which associated with other females most similar in ‘genetical and social background, age, hierarchical position and social class’ (de Waal and Luttrell 1986). In wild Assamese macaques (*Macaca assamensis*), personality similarity was important for bond formation and maintenance (Ebenau et al. 2019). Homophily shapes interactions in other primates (*Colobus guereza,* Kutsukake et al. 2006), as well as zebras (*Equus grevyi,* Sundaresan et al. 2007), meerkats (*Suricata suricatta,* Madden et al. 2011), sperm whales (*Physeter macrocephalus,* Cantor et al. 2015) and common bottlenose dolphins (Lusseau and Newman, 2004).

The social lives of female Indo-Pacific bottlenose dolphins in Shark Bay, Australia, feature a dynamic, fission–fusion grouping pattern that revolves to some degree around maternal kin in an open social network (Mann and Smuts 1998; Frère et al. 2010; Tsai and Mann 2013). Relatedness does not appear to be the sole prerequisite for the formation of social bonds as strong associations also occurred among unrelated females (Frère et al. 2010). Many females engage in foraging strategies that are passed on culturally through the maternal line. Some individuals specialise in ‘sponging’, a culturally transmitted foraging innovation in which primarily female dolphins use marine sponges as tools to flush prey hiding in or on the substrate (Smolker et al. 1997; Krützen et al. 2005, 2014; Patterson and Mann 2011). In the eastern gulf of Shark Bay, sponging dolphins preferentially clustered within mixed-foraging groups, and associations were influenced by sex and matrilineal relatedness, as estimated through behavioural observations (Mann et al. 2012). In the western gulf of Shark Bay, vertical cultural transmission of sponging was quantitatively confirmed through network-based diffusion analyses (Wild et al. 2019). Moreover, genetic structure in the western gulf community appears to have been at least partly driven by the cultural transmission of sponging (Kopps et al. 2014a).

Social homophily among male sponging dolphins has been documented in western Shark Bay (Bizzozzero et al. 2019), but a comprehensive assessment of female associations and community structure has yet to be completed. The presence of a heterogeneous habitat, genetic structure and culturally transmitted foraging strategies provide an ideal opportunity to test to what degree environmental, genetic and/or cultural factors influence dolphin association patterns and thus, sociality. Here, we investigated female dolphin community structure in the western gulf of Shark Bay, accounting for biparental relatedness, matrilineal haplotype-sharing, foraging technique and water depth as a habitat proxy. We utilised the powerful combination of photographic, genetic and behavioural data to identify individuals and track their long-term relationships, methods which are proving invaluable in furthering our understanding of marine mammalian ecology (Connor and Krützen 2015; Allen et al. 2017; King et al. 2021).

## Materials and methods

### Study site and data collection

**Fig. 1:**
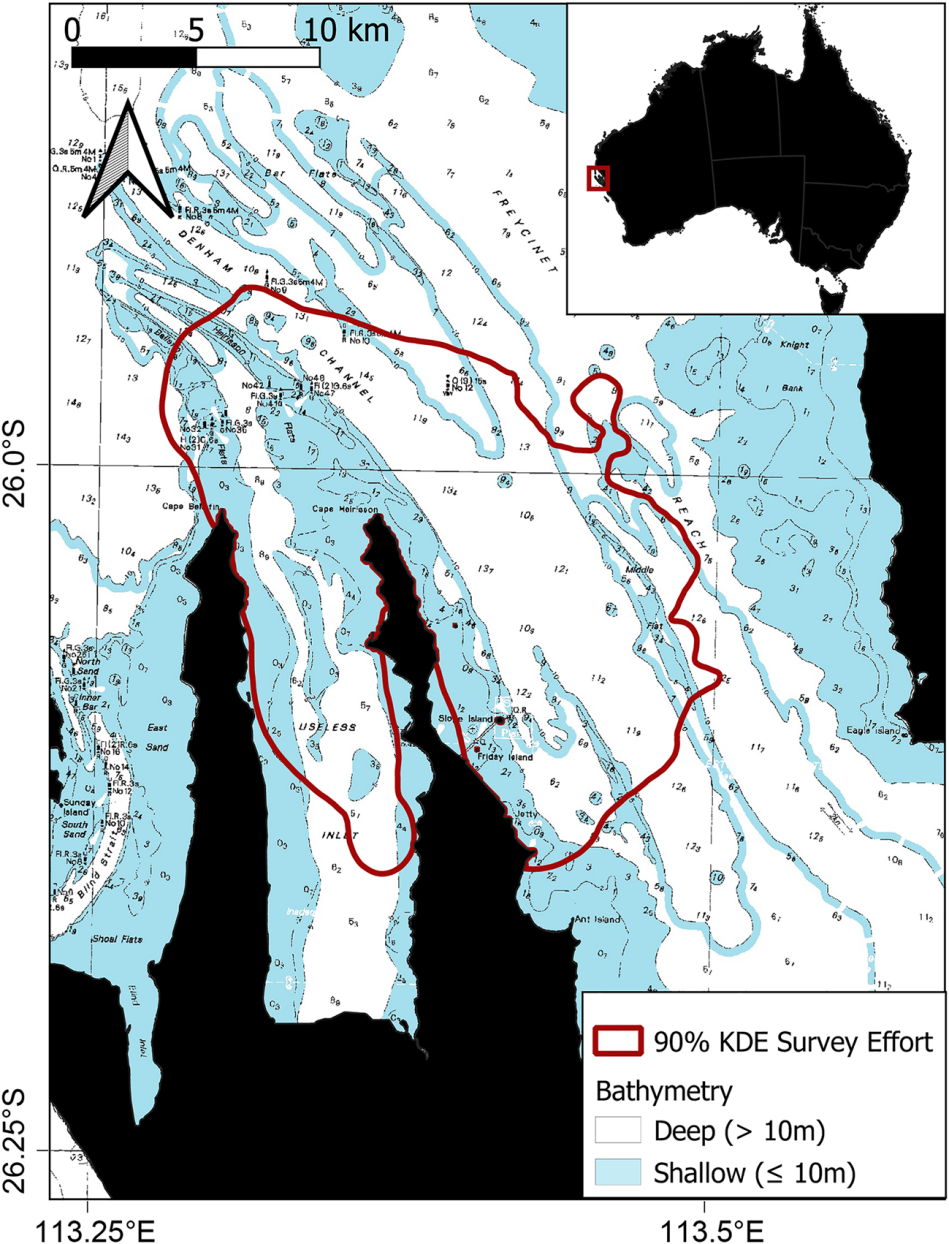
Study area in the western gulf of Shark Bay, Australia. The red polygon depicts the core study area. Deep areas (> 10 m) are shown in white and shallow regions (< 10 m) are shown in blue

**Fig. A1:**
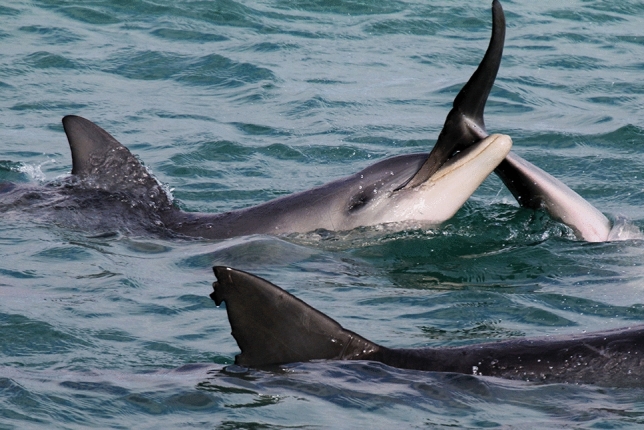
Indo-Pacific bottlenose dolphins (*Tursiops aduncus*) socializing. Distinct markings of the dorsal fin allow systematic photo-identification of each individual (photo credit: Simon Allen)

We collected behavioural and genetic data on Indo-Pacific bottlenose dolphins in western Shark Bay (Fig. 1) during the austral winters of 2007–2019 by conducting boat-based surveys (cf. Bizzozzero et al. 2019), along with the systematic photo-identification of individuals according to the shape, marks, nicks and scars on their dorsal fins (Würsig and Würsig 1977; Nicholson et al. 2012; Appendix Fig. A1). Group membership was determined using the 10-m chain rule (Smolker et al. 1992). For each group encountered, we recorded GPS position, group size and composition, as well as predominant group activity (travel, rest, forage or social, Ethogram in Supplementary Material). We classified an individual as a ‘sponger’ if it had been observed foraging with a sponge on at least two separate days (Mann et al. 2008; Kopps et al. 2014b).

We ascertained an individual’s sex either behaviourally by presence of a dependent calf in the characteristic infant-position for females (Mann and Smuts 1998), observation of the genital area, or genetically. Biopsy samples were collected on an opportunistic basis via remote biopsy sampling (Krützen et al. 2002; see detailed procedures in Supplementary Material). We determined mitochondrial DNA haplotypes and genotyped individuals at 27 microsatellite loci (Krützen et al. 2001, 2004; Nater et al. 2009). All laboratory procedures and PCR protocols are summarised in the Supplementary Material.

To characterise social structure, we implemented the ‘gambit of the group’ approach, which relies on the assumption that individuals are associates when observed in the same group (Whitehead and Dufault 1999). For this study, we focused on females only and excluded dependent calves up to weaning age (≤ 4 years, Mann et al. 2000) to avoid positively biased association indices as a result of including mother-calf dyads. We analysed social structure on two levels, the community and the dyadic level. For the community level analyses, we only included females seen at least ten times in association with at least one other dolphin (*n* = 75). The rationale behind this threshold was that the community assigning algorithm (Blondel et al. 2008) forces all individuals, even those that are primarily solitary, into a community, questioning the biological validity of the approach in such cases. On the dyadic level, we carried out two analyses. To have consistent data sets between both levels, we included in the first analysis the same females as in the community level analysis, i.e., those seen at least ten times in association with at least one other dolphin. In a second analysis, we included individuals seen at least ten times irrespective of whether in association with others or not, following previous work on dyadic associations in dolphins that have identified a threshold of 10-11 sightings to provide robust social networks (e.g., Wiszniewski et al. 2009; Genoves et al. 2018; Bizzozzero et al. 2019; Wild et al. 2020), we repeated the dyadic level analyses with all individuals seen at least ten times irrespective of association. As the results of both dyadic analyses were very similar (Supplementary Material), we report only the first dyadic analysis here.

Given the high identification rate within surveys in our study, we used the Simple Ratio Index (SRI; Ginsberg and Young 1992; Hoppitt and Farine 2018) to measure associations among individuals. If two animals could not physically associate because of a lack of demographic or geographic overlap, the SRIs between these two animals were coded as “not applicable”. To estimate geographic overlap, home ranges were defined using kernel density estimates (Worton 1989) and overlap of individual 95% home ranges were estimated using the method of volume intersection. The SRIs of dyads with less than 25% overlap were coded as “not applicable”.

Genetic samples were available for 58 of the 75 females meeting the inclusion criteria (of a minimum of ten sightings in association) for both mtDNA and nuclear DNA. For the relatedness analyses, individuals for which no genetic data were available were coded as “not applicable”.

A simple yet highly appropriate proxy for habitat type in the western Shark Bay study area is depth below and above the 10-m contour (Tyne et al. 2012). Shallow (< 10 m) areas contain sand flats and seagrass meadows; and deep (10–17 m) water channels have predominantly sandy/rocky substrates and sponge gardens (Tyne et al. 2012). In this study, we approximated habitat exclusively with water depth. We classified a female as occupying either a ‘shallow’ or ‘deep’ habitat based on the mean water depth of all her sightings (< 10 m ‘shallow’, ≥ 10 m ‘deep’). We calculated a pairwise absolute depth difference (in m) as a continuous approximation of habitat similarity, *i.e.*, the smaller the depth difference between two individuals, the more similar the habitat.

### Statistical analyses

We investigated the following four potential correlates that might have a bearing on female sociality: biparental relatedness, matrilinear haplotype, foraging technique (sponger or non-sponger) and habitat proxied by water depth. First, we tested the influence of each factor on the dyadic level and, subsequently, we assessed which of these factors might play a role in community subdivision.

#### Potential factors influencing female sociality at the dyadic level

To quantify the extent to which social bond strength, as measured by the SRI (Ginsberg and Young 1992), i.e., the discrete proportion of the number of surveys in which two animals were observed together over the total number of surveys in which either was observed, can be expressed as a function of a dyad's: (1) biparental relatedness, (2) haplotype identity (same vs. different haplotype), (3) foraging identity (both spongers, both non-spongers or different) and (4) habitat similarity (difference in mean water depth), we fitted a (Bayesian) zero-inflated binomial Generalised Linear Mixed-effects Model (GLMM). To account for the dyadic nature of our data, we incorporated the respective animal IDs as a multi-membership random effect in the model (Hart et al. 2021). We ensured non-collinearity of all four factors by running diagnostics including variance inflation factor (VIF) scores, as well as investigating trace-plots and effective sample size (ESS) estimates.

Model parameters were estimated by allowing four independent Monte Carlo Markov Chains to run for 4000 iterations, with the first half used to ‘warmup’ the algorithm, and the second half to sample the posterior distribution. To aid model convergence we specified weakly regularising priors, and chose normal distributions (*μ* = 0, *σ* = 10 and *μ* = 0, *σ* = 5, respectively) for fixed intercepts and slopes, and a Cauchy distribution (*x*_0_ = 0, *γ* = 2) for the multi-membership random intercept. Chain mixture, convergence and stationarity were confirmed by visually inspecting trace plots and insisting on $\widehat{R}$= 1.00 for all parameters. To achieve this, the value of the ‘adapt_delta’ argument in the ‘brm()’ function was increased to 0.99, and we allowed a maximal tree depth of 15. Overall model performance was assessed by graphical posterior predictive checks, and by calculating a Bayesian version of the *R*^2^-statistic (Gelman et al. 2019). We repeated the zero-inflated binomial GLMM analysis for females seen at least ten times irrespective of association according to the aforementioned dataset specifications (Supplementary Material).

To account for potential overall differences in sociality between spongers and non-spongers, we investigated whether the closest associate of an individual had the same foraging strategy by applying a Bonferroni-corrected binomial test, based on the proportions of spongers and non-spongers in the population of this dataset.

#### Potential factors influencing female sociality at the community level

We performed community structure analyses based on SRIs using the multilevel community detection algorithm, a heuristic method which uses a modularity maximisation approach (Blondel et al 2008) and performs best for ‘small’ networks (*N* ≤ 1000; Yang et al. 2017). We then tested whether biparental relatedness, haplotype identity, foraging identity or habitat correlated with community subdivision using permutation procedures. To achieve this, we randomly assigned the observed biparental relatedness values to the different communities (according to the observed community sizes) 10,000 times and compared the permuted means to the mean observed relatedness values of our study population. We subsequently permuted mitochondrial haplotype identity of dyads (1 for shared, 0 for different mtDNA haplotype) within and between communities with a binomial randomisation 10,000 times. The probability values in the binomial randomisation were set according to the observed haplotype proportions. After each iteration, the mean randomised haplotype identity was calculated for within and between communities and then compared to observed values. Similarly, we permuted foraging identity of dyads (1 for sponger-sponger and non-sponger-non-sponger, 0 for different) within and between communities with a binomial randomisation 10,000 times. The probability values in the binomial randomisation were set according to the observed within/between foraging identity proportions.

Finally, for the habitat, instead of permuting habitat similarity (water depth difference) of dyads, we assigned the mean depths of all individuals to the different communities 10,000 times, taking their original sizes into account, and calculated the standard deviation of depths per community for each permutation. We then compared the permuted standard deviation values to the observed. A smaller observed than randomised standard deviation of water depth within communities indicates a more similar habitat.

We obtained two-sided p-values for all community permutation analyses as follows: the number of permutation values, i.e., mean permuted biparental relatedness per community, mean permuted haplotype similarity, mean permuted foraging similarity and permuted standard deviation of depth per community, that were equal to or higher than the observed values were multiplied by 2 and then divided by the number of permutations.

All analyses were conducted in R version 4.0.4 (R Core Team 2021). We used the asnipe package (Farine 2013) to calculate association indices and social metrics, adehabitatHR package (Calenge 2006) to estimate home ranges and volume of intersection, the brms package (Bürkner 2018) to fit (Bayesian) GLMMs and, finally, the igraph package (Csardi and Nepusz 2006) to perform the community analyses and network visualisation plots.

## Results

### Factors influencing female sociality at the dyadic level

**Fig. 2:**
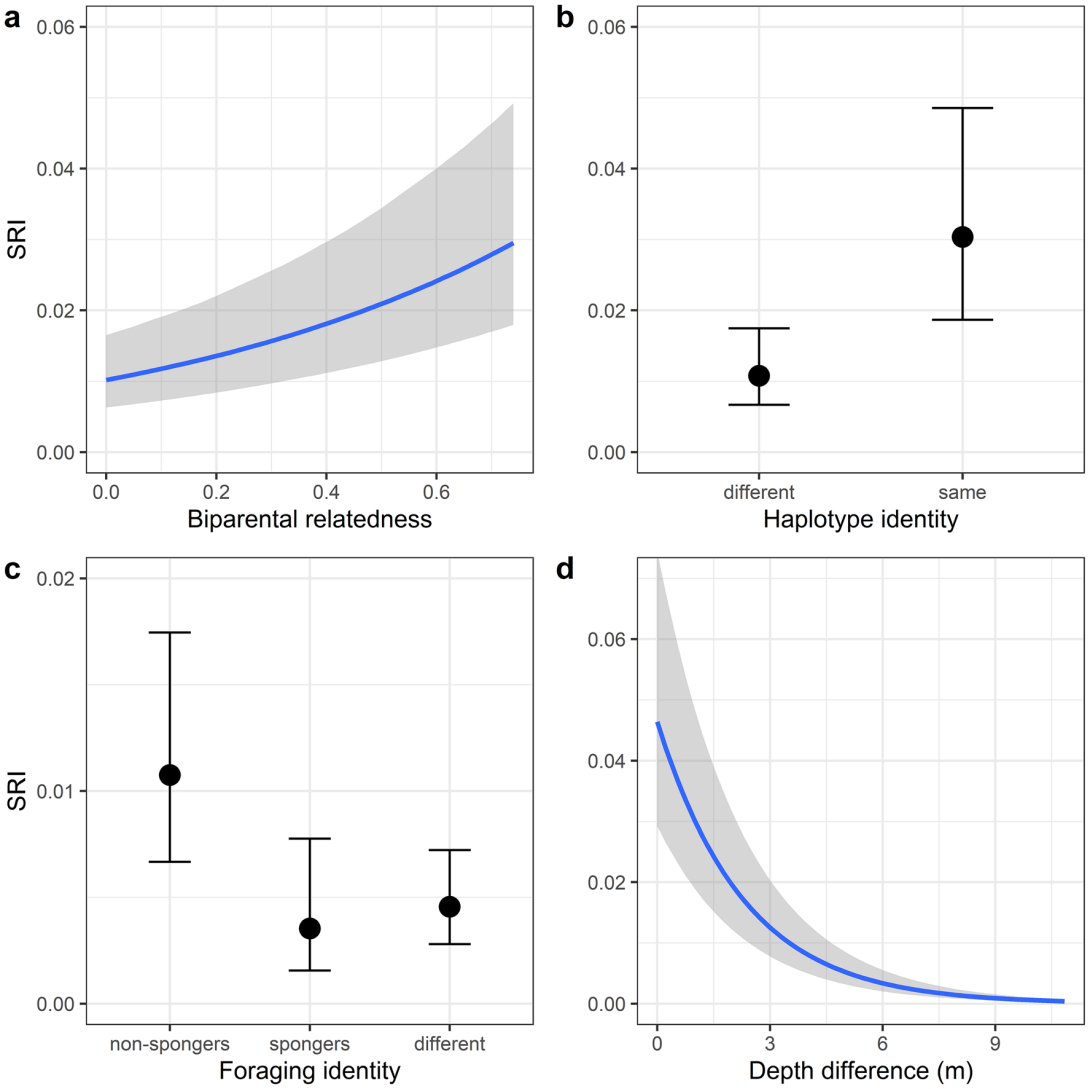
(Bayesian) zero-inflated binomial Generalised Linear Mixed-effects Model (GLMM) of all individuals seen at least ten times in association (*n* = 75). Predicted association index (SRI) values as a function of **a** biparental relatedness, **b** haplotype identity, **c** foraging identity and **d** depth difference. Light grey shaded areas and error bars indicate 95% credible intervals

**Fig. 3:**
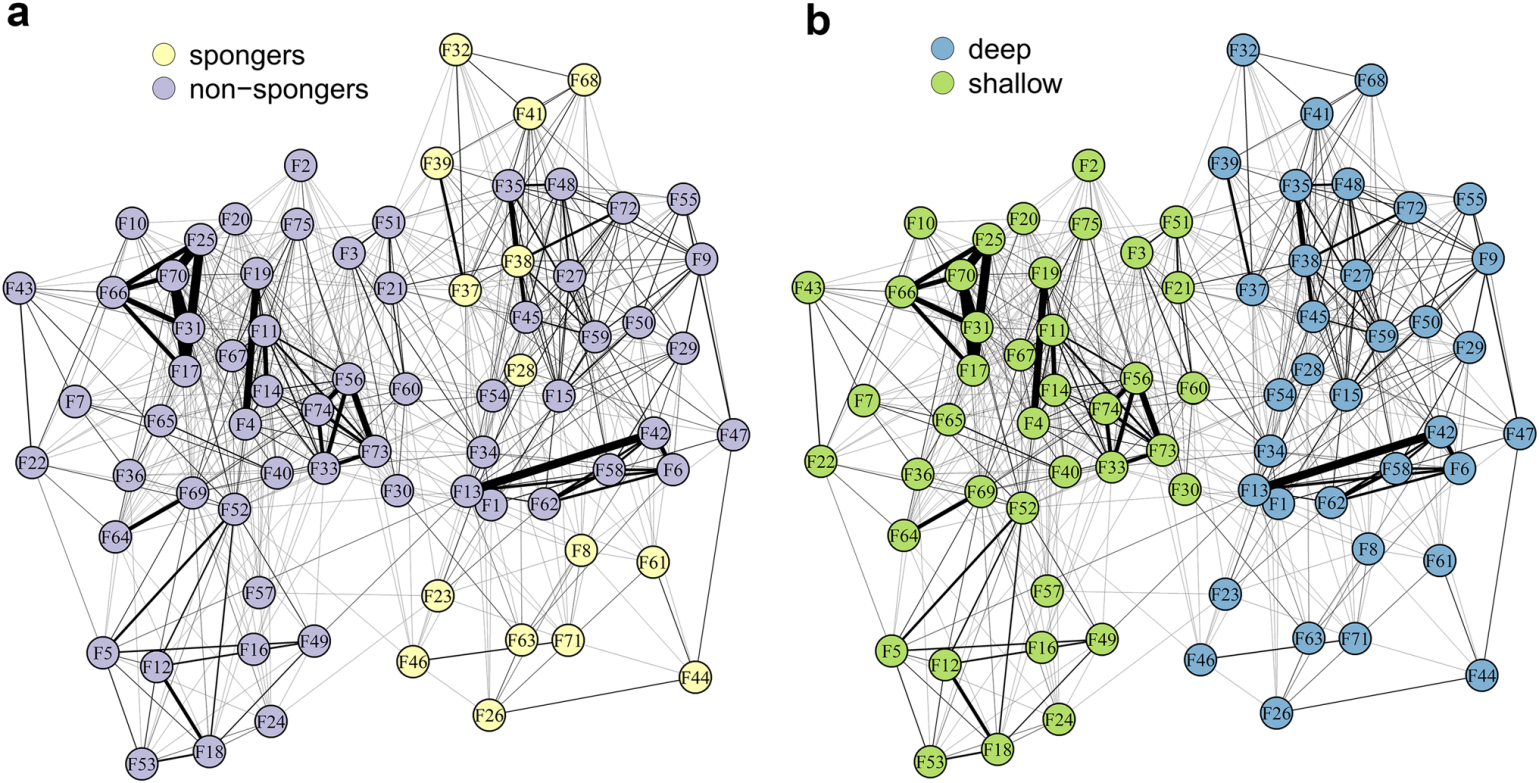
Social network plot colour-coded for **a** foraging strategy (i.e., non-spongers and spongers) and **b** habitat category (i.e., deep and shallow water habitat). Each node represents one individual (*n* = 75). Thickness of edges represents association strength (SRI)

Our zero-inflated binomial GLMM (*R*^2^_Bayesian_: mean = 0.581, 95%-CI = 0.546–0.614) revealed that the odds of two individuals being seen together, *i.e.,* having stronger dyadic social bonds, increased with increasing biparental relatedness (odds ratio = 4.25, 95%-CI = 3.35–5.45, Fig. 2a) and the same haplotype identity (odds ratio = 2.83, 95%-CI = 2.58–3.11, Fig. 2b). Nevertheless, several unrelated dyads also showed high SRI values of up to 0.37. Social bond strength was also affected by foraging identity (Fig. 2c, network visualised in Fig. 3a). Post-hoc pairwise comparisons (using Tukey’s correction for multiple testing) indicated that the odds of a dyad comprising two non-spongers were higher than for a mixed non-sponger-sponger dyad (odds ratio = 2.38, 95%-CI = 1.30–3.60). In contrast, the odds of pure non-sponger and sponger dyads did not differ (odds ratio = 3.07, 95%-CI = 0.87–6.79), and neither did sponger dyads from mixed non-sponger-sponger dyads (odds ratio = 0.77, 95%-CI = 0.44–1.22). Furthermore, the odds of two individuals associating decreased with decreasing habitat similarity (i.e., the odds decreased with increasing difference in water depth: odds ratio = 0.64, 95%-CI 0.62–0.67, Fig. 2d, network visualised in Fig. 3b). Last, the model confirmed that our data were indeed zero-inflated (zero-inflation intercept: mean = 0.11, 95%-CI = 0.08–0.15), emphasizing that not all individuals within our study population that, in principle, could associate, did so. The model findings remain consistent, irrespective of including individuals seen at least ten times, or ten times in association (Supplementary Material).

Spongers had other spongers as closest associates 87% of the time, while 95% of non-spongers had other non-spongers as their closest associates (Fig. 3a). Controlling for the proportion of spongers within the population, both spongers and non-spongers exhibited clear preferences for others with the same foraging technique as their closest associates (Binomial test, *p*_spongers_ < 0.001, *p*_non-spongers_ < 0.001).

### Factors influencing female sociality at the community level

**Fig. 4:**
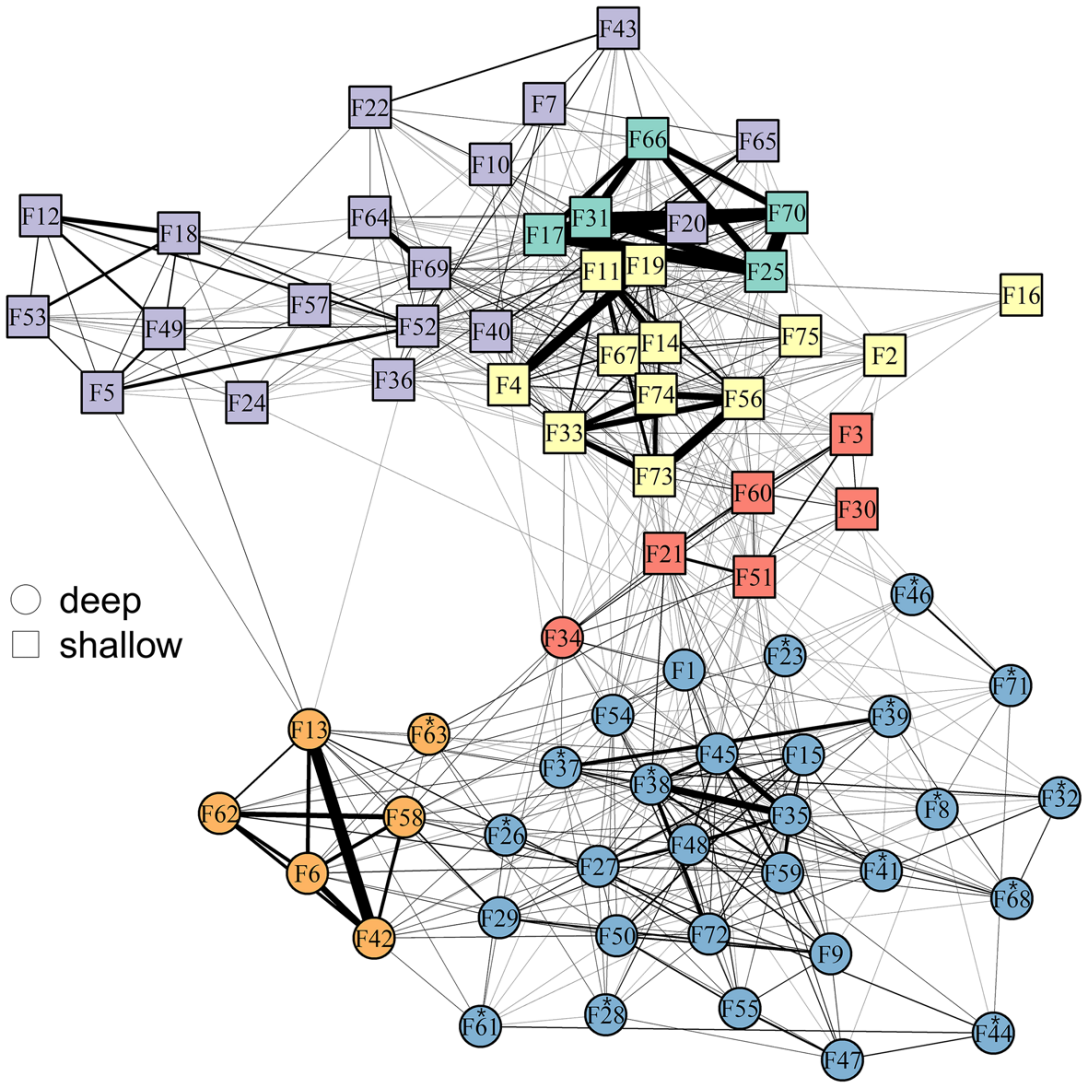
Community network plot. The six different communities are represented by different colours. Each node corresponds to one individual. Node shape represents habitat, with squares referring to shallow-habitat individuals and circles referring to deep-habitat individuals. Sponging individuals are annotated with an asterisk

Our findings at the community subdivision largely mirrored those from our analysis of dyadic associations. Relatedness (both bi- and uniparental), as well as shared foraging identity and habitat similarity were higher within than between communities. The multilevel community detection algorithm (Blondel et al. 2008) yielded six communities among 75 females (Fig. 4). The corresponding modularity score of 0.61 greatly exceeded the threshold of 0.3, suggesting the community division to be meaningful (Newman 2004). Five of the six communities were completely homogenous in terms of habitat similarity (Fig. 4). Except for one individual, spongers clustered within one community which also contained non-sponging individuals.

**Fig. 5:**
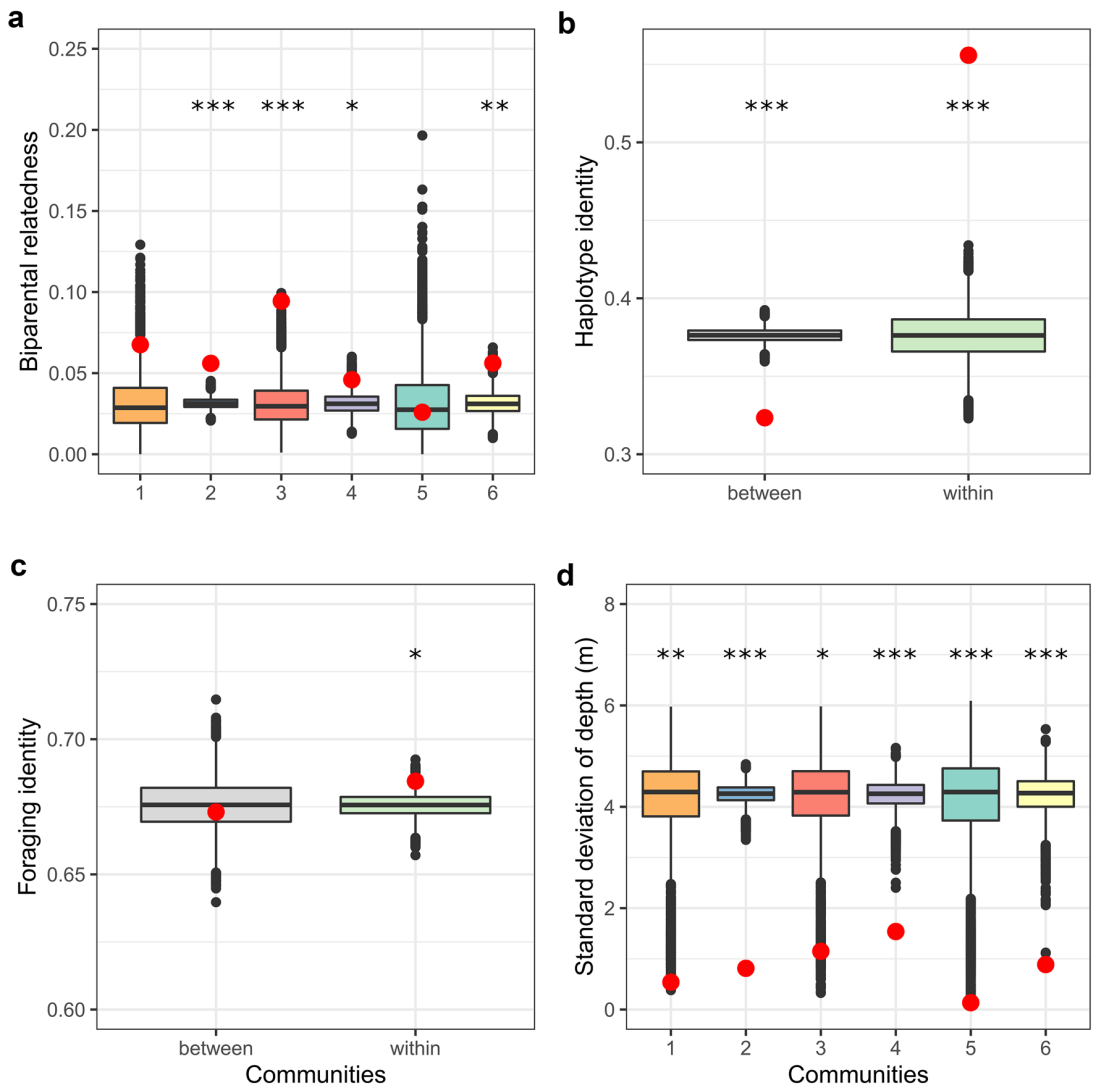
Permutation results of community structure. Observed values are shown as red dots. 10,000 permutations were performed for each factor influencing community structure. **a** Biparental relatedness within communities, **b** haplotype identity between and within communities, **c** foraging identity between and within communities, and **d** standard deviation of depth within communities. The colours used for the different communities in subplots a and d correspond to the colours used in Fig. 4. The orange (1) and blue (2) communities contain spongers. ***p < 0.001, **p < 0.01, *p < 0.05. The horizontal black lines indicate medians and the black vertical lines depict the 1.5×  interquartile range

Biparental relatedness was higher than expected in four of six communities (Fig. 5a). Dyads sharing the same matrilineal haplotype were found more often within the same community than expected by chance (permuted mean haplotype identity within communities: 0.36, observed mean haplotype identity within communities: 0.57, *p* < 0.001, Fig. 5b). Similarly, the distribution of foraging identity within and between communities was not random; dyads sharing the same foraging strategy were more often found within communities than expected by chance (permuted mean foraging identity within communities: 0.67, observed mean foraging identity within communities: 0.69, *p* < 0.05, Fig. 5c).

Depth difference values between individuals ranged from 0 to 12.1 m, with an overall mean of 4.8 m (SE ± 0.9 m). The standard deviation of mean depths was significantly smaller than expected by chance for all six communities (*p* < 0.05), suggesting that communities cluster according to habitat (Fig. 5d). This was further represented in the social network plot, which showed that individuals predominantly associated with others from the same habitat (Fig. 4).

## Discussion

The aim of this study was to investigate the factors that might influence female social structure at both the dyadic and community level in western Shark Bay’s Indo-Pacific bottlenose dolphin population. We found dyadic association patterns and community structure in female bottlenose dolphins were correlated with multiple factors, including biparental relatedness, shared matrilineal haplotype, culturally transmitted foraging technique, and habitat.

We ensured that all individuals of this study could, in principle, associate by carefully filtering the data set so that only dyads with considerably overlapping home-ranges, as well as overlapping lifespans, were considered. Zero-inflation and significant modularity score clearly indicated that associations were non-random. The heterogeneity of association patterns within populations has been reported for other delphinids and primates (Phillips 1998; Pepper et al. 1999; Beck et al. 2012; Titcomb et al. 2015; Baniel et al. 2016).

Associations were generally more common among individuals that were biparentally more closely related and shared the same maternal haplotype. This corroborates previous work by Frère et al. (2010) in eastern Shark Bay, where pairs of associating females exhibited significantly higher biparental relatedness than females under random association patterns. The higher levels of biparental female relatedness in eastern Shark Bay is most likely the result of limited female dispersal (Krützen et al. 2004; Tsai and Mann 2013), allowing the formation of close and persistent social bonds among females. Similar results were also documented in Port Stephens, eastern Australia, where Möller et al. (2006) showed that mean biparental relatedness was significantly higher among frequently associating female Indo-Pacific bottlenose dolphins. Furthermore, increased associations with related individuals were also linked to social network cohesiveness in this population (Wiszniewski et al. 2010). Kin associations among females might be beneficial for protection from predators and male harassment (Wrangham 1980; Connor et al. 2000). Furthermore, assistance in raising and protecting offspring by related females might lead to inclusive fitness benefits (Hamilton 1964).

Although biparental relatedness predicted associations, it did not do so exclusively. We observed strong associations between unrelated dyads, indicating that social relationships among unrelated females may also be important agents of sociality in Shark Bay’s dolphins. In chimpanzees, females preferred kin in most cases, but there were also affiliative relationships among unrelated females linked to dominance rank and sex of their offspring, suggesting that female chimpanzees invest in social relationships with possible adaptive value to themselves and their offspring (Foerster et al. 2015).

Whether dolphins engaged in the foraging technique sponging also had a significant influence on sociality, in that female spongers appeared less social overall, as previously shown in the eastern gulf of Shark Bay (Mann et al. 2008, 2012). Sponging is a largely solitary activity that is considerably more time-consuming than other foraging strategies and, therefore, unlikely to be compatible with high sociality (Mann et al. 2008; Kopps et al. 2014b). Nevertheless, spongers almost always had other spongers as their closest associates. Distinct foraging tactics also influenced social structure in killer whales. Here, prey choice predicted differences in association patterns and sociality: Mammal-eating transient killer whales formed fewer stable associations than fish-eating resident killer whales, which spent considerably more time engaging in social behaviours (Bigg 1987; Morton 1990).

Only recently has the potential effect of habitat complexity on social structure come into focus (He et al. 2019). Indeed, social network position in a wild deer population was shaped by two-dimensional landscape location and pairwise space sharing, indicating that the fine-scale surrounding environment and factors including resource distribution, microclimate, as well as landscape architecture influenced social structure in this ungulate (Albery et al. 2021). Therefore, accounting for influences of the physical habitat when addressing issues regarding social ecology and evolutionary traits of social animals is paramount (Heithaus and Dill 2002; He et al. 2019).

In our study, social interactions between females across habitat types were very limited. Similar habitats, approximated by small differences in mean water depths, strongly predicted dyadic association strength of female dolphins. This suggests that individuals predominantly associate with others that share their habitat preferences. There are two possible explanations as follows: individuals may conform to habitat preferences of close associates or individuals choose their associates from within their preferred habitat. We cannot clearly disentangle these two potential explanations for the observed association patterns. However, previous work revealed that bottlenose dolphins clearly show fine-scale habitat preferences (Allen et al. 2001), which appear to be vertically socially transmitted from mother to offspring. These habitat preferences can be so pronounced that they are reflected in fine-scale genetic patterns of mitochondrial DNA (Sellas et al. 2005; Möller et al. 2007; Kopps et al. 2014a), all pointing towards preferred associations based on established habitat preferences.

Biparental relatedness, shared maternal haplotype and foraging technique, as well as habitat similarity significantly predicted female dyadic associations, indicating that, apart from genetic determinants, homophily may help explain association patterns of female bottlenose dolphins in western Shark Bay. Such heterogeneous association patterns had previously been described based on attributes such as relatedness (Hamilton 1964), age (Wey and Blumstein 2010), reproductive state (Sundaresan et al. 2007; Möller and Harcourt 2008; Diaz-Aguirre et al. 2020), demographic changes (Cantor et al. 2012; Gerber et al. 2020), or behavioural phenotypes (Croft et al. 2009; Ansmann et al. 2012; Mann et al. 2012; Bizzozzero et al. 2019).

We found six distinct communities of female dolphins in our western Shark Bay study area. Haplotype identity was higher within communities than would be expected by chance, a pattern previously described in long-tailed macaques (*Macaca fascicularis*, Ruiter and Geffen 1998), bushbuck antelopes (*Tragelaphus scriptus*, Wronski and Apio 2006) and orang-utans (*Pongo pygmaeus*, Arora et al. 2012), in which mature daughters remain close to their mothers, creating social units of biparental and uniparentally related individuals. Our finding that biparental kinship and shared mtDNA haplotype explained the structure of some of the communities indicated the presence of matrilineal structure where female offspring, although weaned, might remain in the same community with their mothers (e.g., Archie et al. 2006; Wronski and Apio 2006; Gero et al. 2008; Arora et al. 2012; Zanardo et al. 2018).

The low cost of locomotion in dolphins, coupled with a high population density and fission–fusion grouping dynamics, ensures that individuals will interact with many others on a daily basis, including relatives and non-relatives, in different combinations (Smolker et al. 1992; Connor et al. 2000). Specifically, the key factor explaining the range of social bonds in male and female bottlenose dolphins may be the same: the rate that individuals encounter others when they are not with preferred associates (Connor and Whitehead 2005). A male away from his closest allies is likely to encounter other males and a female away from her matrilineal kin is likely to encounter other females. For females, this may disfavour the formation of strict matrilineal groups, as are found in many cercopithecine primates and some toothed whales, in favour of bonds of varying strength with a mixture of female relatives and non-relatives (Connor et al. 2000; Kapsalis 2004; Gouzoules and Gouzoules 2008).

We also assessed whether shared cultural behaviour correlated with community structure. With but one exception, all female spongers were restricted to one community. Our findings are consistent with previous work indicating that shared culturally transmitted foraging specialisations affect social structure in a Lahilles' bottlenose dolphin (*T. t. gephyreus*) population in southern Brazil (Machado et al. 2019). Dolphins that specialise in a foraging technique that involves coordinating with shore-based fishers preferentially associate with others using the same strategy, and this was also true for associations extending beyond foraging contexts (Machado et al. 2019). Sympatric sperm whale clans provide another example of cultural behaviour influencing sociality, differing in their vocal, movement, foraging and social behaviours (Cantor and Whitehead 2015; Cantor et al. 2015). In the eastern gulf of Shark Bay, dolphins display a degree of foraging homophily where spongers, although generally more solitary, behaved more ‘cliquishly’ in that they grouped together more cohesively (Mann et al. 2012). However, individuals did not cluster uniquely according to their foraging specialisation. Spongers clustered together with a few non-spongers within two of six clusters (Mann et al. 2012). Homophily in sponging behaviour likewise appears to influence alliance composition of male dolphins in western Shark Bay (Bizzozzero et al. 2019). It appears that cultural processes also contribute to intrapopulation variation in social structure in female dolphins.

Finally, habitat characteristics appear to have a bearing on dolphin community structure in the western gulf of Shark Bay. Community subdivision in connection with distinct water depths was also reported in a *T. aduncus* population within a lagoon in the Indian Ocean archipelago of Mayotte, where the authors reported one shallow-water community and a second community close to a deep reef bank (Kiszka et al. 2012). Similarly, female dolphin communities within the western gulf of Shark Bay are habitat specific, indicating that habitat plays a pivotal role for the population’s social structure (Kopps et al. 2014a).

## Conclusions

As in several other taxa, extrinsic factors in combination with intrinsic behaviours appear to shape association patterns and sociality in a society with a dynamic fission–fusion grouping pattern. Specifically, biparental and shared mitochondrial haplotypes, habitat similarity, and shared, vertically culturally transmitted foraging behaviour affected social structure in female Indo-Pacific bottlenose dolphins. While maternal as well as biparental kinship suggested some degree of matrilineal structure, social associations among unrelated individuals were also documented. This range of relationships may owe to the low cost of locomotion and therefore the potential for individuals to encounter preferred as well as non-preferred social partners. Two female communities consisted of sponging and non-sponging individuals, suggesting that although associations are higher among individuals sharing the same foraging behaviour, this was not exclusively so. Cross-habitat dyadic associations were rare and female communities appeared to be habitat-specific. Sociality among female Indo-Pacific bottlenose dolphins is thus likely influenced by a complex combination of genetic aspects, cultural processes and environmental factors.

### Supplementary Information

Below is the link to the electronic supplementary material.

Supplementary file1 (PDF 394 KB)

Supplementary file2 (TXT 6 KB)

Supplementary file3 (R 21 KB)

Supplementary file4 (XLSX 1781 KB)

Supplementary file5 (PDF 209 KB)

## Data Availability

The datasets generated for and analysed during the current study together with the R-scripts to replicate analyses and Figures are available in the Supplementary Material.
