# Supplementary material for: Association patterns and community structure among female bottlenose dolphins: environmental, genetic and cultural factors
Source: Mamm Biol. 2022 Nov 2;102(4):1373–87. doi: 10.1007/s42991-022-00259-x (PMC10040398; doi:10.1007/s42991-022-00259-x)
Supplement: Supplementary file 1 — Supplementary file1 (PDF 394 KB) [file 42991_2022_259_MOESM1_ESM.pdf]

Supplementary Material

Association patterns and community structure among female bottlenose dolphins: environmental, genetic and cultural factors

Svenja M. Marfurt[1], Simon J. Allen[1,2,3], Manuela R. Bizzozzero[1], Erik P. Willems[4], Stephanie L. King[2,3], Richard C. Connor[5], Anna M. Kopps[6], Sonja Wild[7,8], Livia Gerber[1,6], Samuel Wittwer[1], Michael Krützen[1]

Published in: Karczmarski L, Chan SCY, Chui SYS, Cameron EZ (2022). Individual identification and photographic techniques in mammalian ecological and behavioural research – Part 2: Field studies and applications. Mammalian Biology (Special Issue), 102 (4)
https://link.springer.com/journal/42991/volumes-and-issues/102-4

Author affiliations:

[1] Evolutionary Genetics Group, Department of Anthropology, University of Zurich, 8057 Zurich, Switzerland

[2] School of Biological Sciences, University of Western Australia, Perth, Western Australia 6009, Australia

[3] School of Biological Sciences, University of Bristol, Bristol BS8 1TQ, UK

[4] Department of Anthropology, University of Zurich, 8057 Zurich, Switzerland

[5] Biology Department, UMASS Dartmouth, North Dartmouth, MA, USA

[6] Evolution and Ecology Research Centre, School of Biological, Earth and Environmental Sciences, University of New South Wales, Sydney, New South Wales 2052, Australia

[7] Cognitive and Cultural Ecology Research Group, Max Planck Institute of Animal Behavior, Am Obstberg 1, 78315, Radolfzell, Germany

[8] Centre for the Advanced Study of Collective Behaviour, University of Konstanz, Universitätsstrasse 10, 78464 Konstanz, Germany

Corresponding author:

Svenja M. Marfurt, Email: svenja.marfurt@uzh.ch

1. Genetic analyses

Genetic sampling has been conducted since the establishment of the study site off Useless Loop in western Shark Bay in 2007. Biopsy samples have been collected on an opportunistic basis and consist of skin and blubber tissue (plugs). Biopsy samples are preferably taken from underneath the dorsal fin using the PAXARMS sampling system. It was specifically designed for the purpose of sampling small cetaceans and only has minimal short-term, and no evident long-term effects (Krützen et al. 2002). We stored the collected biopsy samples in saturated NaCl / 20% dimethyl sulphoxide solution (following Amos and Hoelzel, 1991). We performed the DNA extractions using a slightly modified version of the Gentra Purgene mouse tail protocol (Qiagen). To enhance tissue lysis, we added a stainless-steel bead during an overnight incubation at 55 °C in an overhead rotator. If the lysis was not complete after this incubation, we added additional Proteinase K (20 mg / 1 ml). Once tissue was lysed, we added 3 µl RNAse A (Sigma-Aldrich) and incubated at 37°C for 60 min. Instead of isopropanol, we used 100% ethanol to purify the DNA extracts. We measured the concentrations of the extracts using a Nanodrop OneC (Thermo Fisher Scientific) and diluted all samples to a concentration of 20 ng/µl using DNA Hydration Solution (Qiagen). All PCR and sequencing protocols are provided in Table S1.

1.1 Molecular sex determination

We determined sex genetically by amplifying two sex-chromosome specific loci, ZFX and SRY, using the primers P1- 5EZ and P2-3EZ (Aasen and Medrano 1990), as well as Y53-3C and Y53-3D (following the protocol by Gilson et al., 1998). By visualizing the PCR product with a staining agent (GelRed™) on a 1.5% agarose gel using UV-light, we were able to determine sex. As females are homogametic (two X chromosomes), they showed only one band whereas heterogametic males (one X and one Y chromosome) showed two.

1.2 Maternal relatedness (mtDNA haplotype)

To obtain the mtDNA (mitochondrial DNA) haplotype, we amplified a ca. 430 base pair segment of the HVR-I (hypervariable sequence of the control region) using d-loop specific primers (following Baker *et al.*, 1993) with a polymerase chain reaction (PCR). After a clean-up (described in Bacher *et al.*, 2010), the amplified regions were sequenced using a BrilliantDye™ Cycle Sequencing Terminator Kit (Nimagen). Using Sequence Analysis (version 5.3.1, Applied Biosystems) and Unipro Ugene (Okonechnikov et al. 2012), the generated mtDNA sequences were quality controlled, analysed and aligned with the MUSCLE algorithm in Ugene. Every female for which we had an mtDNA sample available was assigned a haplotype (haplotypes D, K, H, F or E).

1.3 Biparental relatedness

We genotyped each individual for 27 microsatellites loci using three different multiplex PCR setups (Appendix Table 1). The nineteen tetranucleotides and eight dinucleotides were amplified using Qiagen Multiplex Kit™. Multiplex 1 contained the following markers: E12, Tur4_66, Tur4_98, Tur4_108, Tur4_111, Tur4_117, Tur4_128 (Nater et al. 2009), and MK6 (Krützen et al. 2001). The following loci were amplified in multiplex 2: D8, F10, Tur4_87, Tur4_91, Tur4_138, Tur4_141 (Nater et al. 2009), and

D22 (Shinohara et al. 1997). Finally, Tur4_80, Tur4_132, Tur4_142, Tur4_153, Tur4_162 (Nater et al. 2009), MK3, MK5, MK8, MK9 (Krützen et al. 2001), EV37 (Valsecchi and Amos 1996), and KWM12 (Hoelzel et al. 1998) were amplified in multiplex 3. PCR conditions are shown in Table S1. We added 1 µl of the 1:75 diluted PCR-products to 10 µl of Hi-Di formamide (Thermo Fisher Scientific) with 0.07 µl size standard (GenScanTM500Liz™,Thermo Fisher Scientific). After four minutes of denaturation at 94°C, the single-stranded PCR products were analyzed by capillary electrophoresis using an ABI 3730 DNA Sequencer (Applied Biosystems). We scored alleles in GeneMapper 4.0 software (Thermo Fisher Scientific). To examine pairwise relatedness, a background population was needed for comparison of the obtained dyadic relatedness values. In this background population, we included all genotyped individuals of western Shark Bay with fewer than 10 missing loci (n = 167). We compared the obtained r-values of the seven different relatedness estimators given by COANCESTRY (Wang 2011) with known mother-daughter pairs. Equally to what Bizzozzero, (2016) established for the western Shark Bay population, we found the triadic likelihood (TrioML) estimator to be the most appropriate.

Table S1: PCR set-up conditions and the corresponding PCR profiles. For molecular sexing, three microsatellite multiplexes (MP1, MP2 and MP3) as well as for the mtDNA-amplification PCR and for the mtDNA cycle-sequence PCR.

PCR Setup **Sexing**	Concentration	Final Conc in PCR	Per rxn [µl]
Template [µl]			1.00
Primer P1-5EZ	10	0.15 [µM]	0.15
Primer P2-3EZ	10	0.15 [µM]	0.15
Primer Y53-3C	10	0.15 [µM]	0.15
Primer Y53-3D	10	0.15 [µM]	0.15
dNTPs	10	0.1 [mM]	0.10
$MgCl_2$	25	0.3125 [mM]	0.125
Buffer (with MgCl2)	10	1 [x]	1.00
Taq polymerase [u/µl]	2.5	0.125 [u]	0.05
ddH_2O			7.125
Final Volume [µl]			10.00

PCR Profile **Sexing**	Temperature [ºC]	Time [min' sec'']	No. of cycles
Initial Denaturation	94	4'	1
Denaturation	94	45''	
Annealing	58	45''	**40**
Extension	72	1'	
Final Extent.	72	10'	1

PCR Setup **MP1/2/3**	Concentration	Final Conc in PCR	Per rxn [µl]
Template [µl]			1.00
Primermix	10	0.1 [µM]	0.80
Mulitplex Mastermix	2	1 [x]	4.00
ddH_2O		[µl]	2.20
Final volume		[µl]	8.00

PCR Profile **MP1/2/3**	Temperature [ºC]	Time [min' sec'']	No. of cycles
Initial Denaturation	95	15'	1
Touchdown 1			
Touchdown 2			
Denaturation	95	30"	
Annealing	60	90"	**30 (34** for MP3**)**
Extension	71	45"	
Final Extent.	60	30'	1

PCR Setup **D-Loop**	Concentration	Final Conc in PCR	Per rxn [µl]
Template [µl]			1.00
Primer dlp 1.5	10	0.1 [µM]	0.10
Primer dlp 5	10	0.1 [µM]	0.10
dNTPs	10	0.1 [µM]	0.10
MgCl2	25	0.3125 [nM]	0.125
Buffer	10	1 [x]	1.00
Taq Polymerase	2.5	0.1 [u]	0.05
ddH_2O			7.53
Final volume			10.00

PCR Profile **D-Loop**	Temperature [ºC]	Time [min’ sec’’]	No. of cycles
Initial Denaturation	94	3’	1
Denaturation	93	45"	
Annealing	48	60”	**39**
Extension	72	90”	
Final Extent.	72	3’	1

Cycle Seq. **D-Loop**	Concentration	Final Conc in PCR	Per rxn [µl]
Template [µl]			1.00
Primer dlp 1.5 or dlp 5	10	0.4 [µM]	0.40
5x CS Buffer	5	3.7 [x]	1.85
ddH20			6.45
Brilliant Dye	2.5	1[x]	0.30
Final volume		[µl]	10.00

Cycle Seq. Profile	Temperature [ºC]	Time [min’ sec’’]	No. of cycles
Initial Denaturation	95	45’’	1
Denaturation	95	30‘’	
Annealing	52	20’’	**30**
Extension	60	2’	
Final Extent.	60	∞	1

2. Ethogram

(From Bizzozzero et al., 2019, Supplementary Information: 2. Ethogram)

During boat-based surveys of dolphin groups, within the first five minutes, we recorded GPS position, environmental parameters (including sea state, water depth and temperature), group size and composition, as well as predominant group activity (rest, travel, forage, socialise, or unknown). The behavioural states were defined according to a long-established protocol used in the field since the founding of this study in 2007, but was itself based on an ethogram developed much earlier still (Smolker et al. 1992). The categories are as follows:

Rest – Dolphins in a tight group, moving very slowly with regular, peduncle or tail-out dives, or snagging – dives and surfacing bouts are often synchronous during resting behaviour. During rest, there is no evidence of foraging or socialising (although juveniles/calves may socialise while adults rest). Resting groups regularly snag en masse.

Travel - Individuals clustered or line abreast and moving in one general direction (i.e., no rapid changes in direction) for a period of several minutes or during consecutive surfacing bouts.

Social – Social behaviour is characterised by body contact, rubbing and petting, often accompanied by splashing, surface activity and acoustic behaviour. Bottlenose dolphins also perform a variety of synchronous behaviours.

Forage - Foraging is one of the most frequently observed behaviours, often involving lone dolphins or widely dispersed groups. An exception to this occurs when one or more dolphins remain in close proximity to a foraging dolphin for social reasons (i.e., when males are herding a female). Both inter-individual geometry (iig) and dive type are important in determining whether or not dolphins are foraging, independent of observations of feeding. Movement and iig are usually characterised by milling or meandering during foraging. In deeper water, foraging usually involves multiple breath surfacing bouts, culminating in a tail-out or peduncle dive. In shallower water, regular bottom grubbing, rapid surfaces and (fish) chases are often observed.

3. Additional analyses

3.1. Datasets

Minimum of ten sightings in association

The mean number of sightings of the 75 individuals seen at least ten times in association was 32 with a minimum of 11 and a maximum of 123 sightings. Of these, 40 females were categorized as shallow- and 35 as deep-habitat individuals. The investigated individuals contained 15 spongers. A total of 30 individuals were assigned haplotype E, 5 haplotype F, 19 haplotype H and 4 individuals haplotype K (Table S2, ids 1-75). To date, 63 of the total 253 identified females in the western gulf of Shark Bay have been classified as spongers which results in a proportion of 0.25.

Minimum of ten sightings irrespective of association

The mean number of sightings of the 122 individuals seen at least ten times irrespective of association was 38 with a minimum of 10 and a maximum of 126 sightings. Of these, 62 were categorized as shallow- and 60 as deep-habitat individuals. The investigated individuals contained 35 spongers (proportion of 0.29). A total of 47 individuals were assigned haplotype E, 5 haplotype F, 24 haplotype H, 4 individuals haplotype K and one individual haplotype D (Table S2, ids 1-122).

3.2 Supplementary methods & results

Mantel test (at least ten times in association)

To determine whether individuals with higher biparental relatedness values (biparental relatedness estimation procedures are summarised above) associated more frequently than unrelated individuals, we tested for a correlation between the biparental relatedness matrix and the SRI-matrix using a permutation-based Mantel test in the R package vegan (Oksanen et al. 2018). We then tested whether maternally related individuals, *i.e.*, those sharing the same mtDNA haplotype, associated more frequently than those with different haplotypes by applying a permutation-based Mantel test on the mtDNA haplotype-identity matrix (coded 1 for a dyad sharing the same haplotype, 0 for a dyad with different haplotypes) and the SRI-matrix. Similarly, in order to assess whether sponging correlated with female dyadic associations, we tested whether SRIs were higher amongst dyads with the same foraging strategy, *i.e.*, among spongers and non-spongers, than expected by chance. Likewise, applying a permutation-based Mantel test in the R-package vegan (Oksanen et al. 2018), we assessed whether SRIs were correlated with habitat similarity.

Biparental relatedness, haplotype-sharing, foraging strategy and habitat correlated with the strength of associations. Dyads with higher biparental relatedness (Mantel statistic $r = 0.24$, $p < 0.001$) and who shared the same haplotype (Mantel statistic $r = 0.19$, $p<0.001$) were more likely to associate. Furthermore, SRI values were also correlated with foraging strategy (Mantel statistic $r = 0.13$, $p<0.001$) and habitat similarity (Mantel statistic $r = 0.20$, $p < 0.001$). Stronger associations were documented almost exclusively among individuals of the same habitat category.

Zero-inflated binomial GLMM (at least ten times irrespective of association)

Our zero-inflated binomial GLMM ($R^2_{Bayesian}$: mean= 0.588, 95%-CI= 0.537 – 0.636) revealed that the odds of two individuals being seen together (*i.e.* have a stronger dyadic social bond, as proxied by the SRI) increased with biparental relatedness (odds ratio= 2.61, 95%-CI= 1.972– 3.427, Fig. S1a), haplotype identity (odds ratio= 1.935, 95%-CI= 1.738 – 2.153, Fig. S1b), and habitat similarity (*i.e.* the odds decreased with increasing difference in mean water depth: odds ratio= 0.635, 95%-CI= 0.612 – 0.658, Fig. S1d). Social bond strength was also affected by foraging identity (Fig. S1c). Post-hoc pairwise comparisons (using Tukey’s correction for multiple testing) indicated that the odds of a dyad comprising two non-spongers were higher than it being a mixed non-sponger-sponger dyad (odds ratio= 3.76, 95%-CI= 2.420 – 5.45) and marginally higher than it being a sponger dyad (odds ratio= 2.89, 95%-

CI= 1.13 – 5.61). The odds of sponger dyads did not differ from mixed non-sponger-sponger dyads (odds ratio= 1.30, 95%-CI= 0.787 – 1.92).

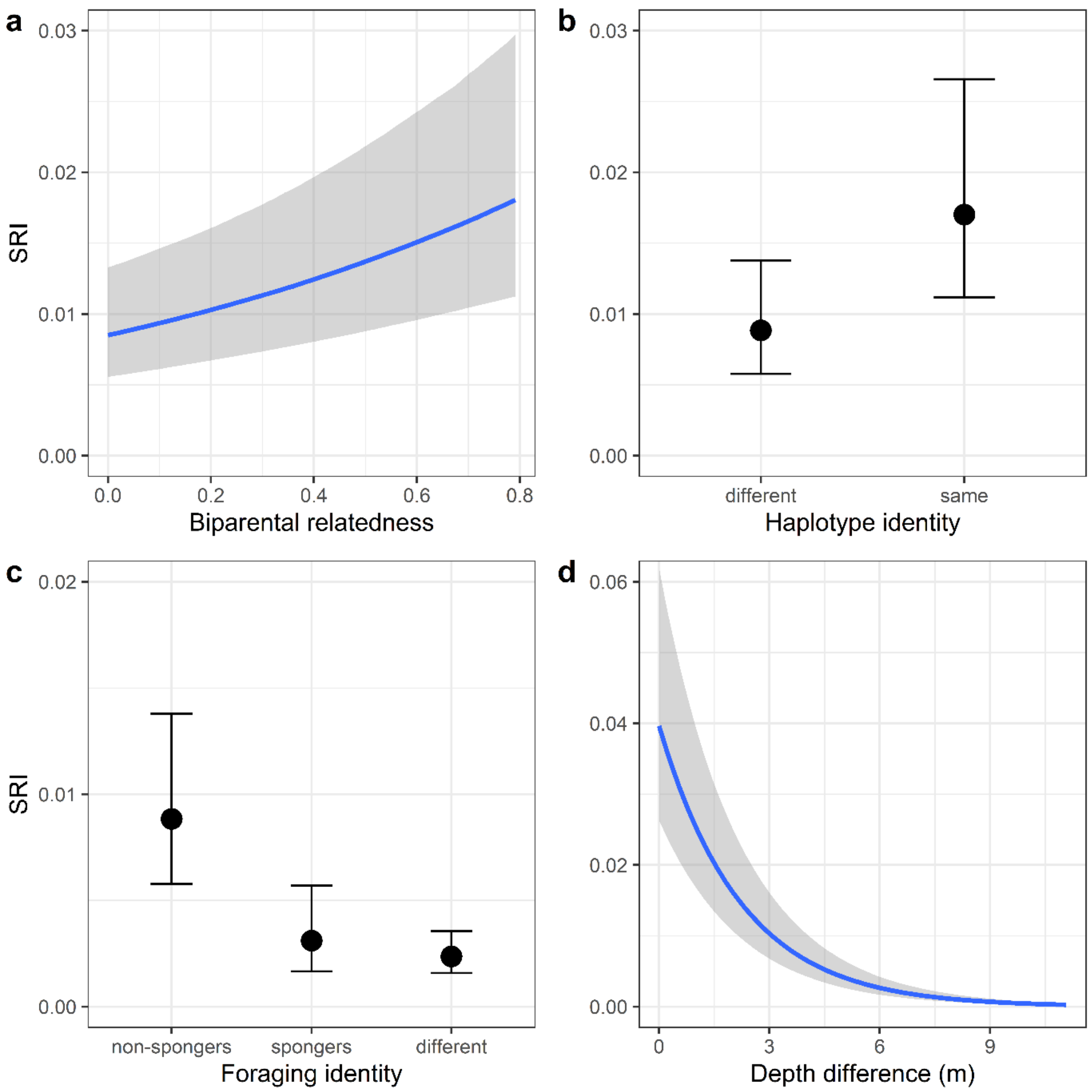

Figure S1: (Bayesian) zero-inflated binomial Generalized Linear Mixed-effects Model (GLMM) for individuals seen at least ten times irrespective of association (n = 122). Predicted association index (SRI) values as a function of (**a**) biparental relatedness, (**b**) haplotype identity, (**c**) foraging identity and (**d**) depth difference. Light grey shaded areas indicate 95% confidence intervals

3.3 Summary of the dataset

Table S2: Attribute data of individuals analyzed in this study. All individuals with confirmed female sex, which were seen at least ten times in association (first 75 ids) and seen at least ten times irrespective of association (ids 76-122), excluding dependent calves. Table of all individual IDs as numbers, the mean depth at which an individual was observed, its habitat category (deep or shallow habitat) and its foraging technique (sponger or non-sponger). The last column shows the community each individual was assigned to in the community level analysis using the Multilevel (ML) algorithm. The colours correspond to the community colours in Figures 3 and 4

id	haplotype	mean depth [m]	forage	habitat	ML community
F1	NA	10.8	Non-sponger	deep	2
F2	H	5.1	Non-sponger	shallow	6
F3	H	5.2	Non-sponger	shallow	3
F4	E	5.1	Non-sponger	shallow	6
F5	E	5.4	Non-sponger	shallow	4
F6	H	10.8	Non-sponger	deep	1
F7	NA	1.9	Non-sponger	shallow	4
F8	E	13.8	Sponger	deep	2
F9	NA	12.8	Non-sponger	deep	2
F10	H	3.1	Non-sponger	shallow	4
F11	E	4.0	Non-sponger	shallow	6
F12	NA	5.6	Non-sponger	shallow	4
F13	NA	10.3	Non-sponger	deep	1
F14	E	3.7	Non-sponger	shallow	6
F15	F	13.0	Non-sponger	deep	2
F16	E	2.7	Non-sponger	shallow	6
F17	NA	3.6	Non-sponger	shallow	5
F18	E	5.8	Non-sponger	shallow	4
F19	E	5.0	Non-sponger	shallow	6
F20	H	2.8	Non-sponger	shallow	4
F21	H	6.7	Non-sponger	shallow	3
F22	K	2.6	Non-sponger	shallow	4
F23	E	11	Sponger	deep	2
F24	E	5.8	Non-sponger	shallow	4
F25	NA	3.4	Non-sponger	shallow	5
F26	E	13.6	Sponger	deep	2
F27	E	12.7	Non-sponger	deep	2
F28	E	12.3	Sponger	deep	2
F29	NA	12.4	Non-sponger	deep	2
F30	H	6.4	Non-sponger	shallow	3
F31	K	3.4	Non-sponger	shallow	5
F32	E	13.6	Sponger	deep	2
F33	F	3.9	Non-sponger	shallow	6
F34	H	8.2	Non-sponger	shallow	3
F35	E	13.5	Non-sponger	deep	2
F36	NA	3.4	Non-sponger	shallow	4
F37	E	13.2	Sponger	deep	2

F38	E	12.9	Sponger	deep	2
F39	E	13.2	Sponger	deep	2
F40	H	2.7	Non-sponger	shallow	4
F41	E	13.0	Sponger	deep	2
F42	NA	10.8	Non-sponger	deep	1
F43	NA	3.5	Non-sponger	shallow	4
F44	E	13.7	Sponger	deep	2
F45	E	13.0	Non-sponger	deep	2
F46	E	11.7	Sponger	deep	2
F47	F	13.5	Non-sponger	deep	2
F48	NA	13.3	Non-sponger	deep	2
F49	NA	5.2	Non-sponger	shallow	4
F50	F	12.1	Non-sponger	deep	2
F51	H	5.4	Non-sponger	shallow	3
F52	E	5.2	Non-sponger	shallow	4
F53	E	5.9	Non-sponger	shallow	4
F54	H	11.5	Non-sponger	deep	2
F55	NA	12.9	Non-sponger	deep	2
F56	H	4.0	Non-sponger	shallow	6
F57	E	6.5	Non-sponger	shallow	4
F58	H	11.7	Non-sponger	deep	1
F59	E	13.5	Non-sponger	deep	2
F60	H	5.5	Non-sponger	shallow	3
F61	E	12.8	Sponger	deep	2
F62	H	11.7	Non-sponger	deep	1
F63	E	11.0	Sponger	deep	1
F64	NA	3.0	Non-sponger	shallow	4
F65	H	2.1	Non-sponger	shallow	4
F66	K	3.6	Non-sponger	shallow	5
F67	NA	5.7	Non-sponger	shallow	6
F68	E	14.0	Sponger	deep	2
F69	H	3.0	Non-sponger	shallow	4
F70	K	3.3	Non-sponger	shallow	5
F71	NA	12.5	Sponger	deep	2
F72	F	12.9	Non-sponger	deep	2
F73	H	4.3	Non-sponger	shallow	6
F74	H	3.6	Non-sponger	shallow	6
F75	E	5.3	Non-sponger	shallow	6
F76	E	13.6	Sponger	deep	
F77	NA	12.0	Sponger	deep	
F78	NA	12.2	Sponger	deep	
F79	D	5.4	Non-sponger	shallow	
F80	NA	12.1	Sponger	deep	
F81	NA	5.5	Non-sponger	shallow	
F82	NA	13.6	Sponger	deep	

F83	E	12.8	Sponger	deep
F84	NA	8.9	Non-sponger	shallow
F85	NA	12.7	Non-sponger	deep
F86	E	12.5	Sponger	deep
F87	NA	4.4	Non-sponger	shallow
F88	NA	8.5	Non-sponger	shallow
F89	E	14.8	Sponger	deep
F90	E	14.4	Sponger	deep
F91	E	9.2	Sponger	shallow
F92	E	13.0	Sponger	deep
F93	NA	14.2	Sponger	deep
F94	NA	12.8	Sponger	deep
F95	E	5.8	Non-sponger	shallow
F96	NA	7.6	Non-sponger	shallow
F97	H	5.5	Non-sponger	shallow
F98	NA	13.5	Sponger	deep
F99	NA	11.4	Non-sponger	deep
F100	NA	4.1	Non-sponger	shallow
F101	E	13.5	Non-sponger	deep
F102	NA	3.6	Non-sponger	shallow
F103	NA	5.7	Non-sponger	shallow
F104	NA	7.2	Non-sponger	shallow
F105	H	13.3	Non-sponger	deep
F106	NA	14.0	Sponger	deep
F107	E	6.8	Non-sponger	shallow
F108	E	7.3	Non-sponger	shallow
F109	E	14.2	Sponger	deep
F110	NA	8.2	Non-sponger	shallow
F111	H	4.5	Non-sponger	shallow
F112	H	5.8	Non-sponger	shallow
F113	E	14.7	Sponger	deep
F114	NA	8.2	Non-sponger	shallow
F115	E	12.9	Sponger	deep
F116	H	4.4	Non-sponger	shallow
F117	NA	13.3	Non-sponger	deep
F118	E	11.0	Sponger	deep
F119	E	11.9	Sponger	deep
F120	E	5.1	Non-sponger	shallow
F121	NA	13.0	Non-sponger	deep
F122	NA	12.5	Sponger	deep

References

Aasen E, Medrano JF (1990) Amplification of the zfy and zfx genes for sex identification in humans, cattle, sheep and goats. Nat Biotechnol 8:1279–1281. https://doi.org/10.1038/nbt1290-1279

Amos B, Hoelzel AR (1991) Long-term preservation of whale skin for DNA analysis. In: Genetic ecology of whales and dolphins. Report of the International Whaling Commission

Bacher K, Allen S, Lindholm AK, et al (2010) Genes or Culture: Are mitochondrial genes associated with tool use in bottlenose dolphins (Tursiops sp.)? Behav Genet 40:706–714. https://doi.org/10.1007/s10519-010-9375-8

Baker CS, Perry A, Bannister JL, et al (1993) Abundant mitochondrial DNA variation and world-wide population structure in humpback whales. Proc Natl Acad Sci U S A 90:8239–8243

Bizzozzero M (2016) Alliance formation and sponge tool use i n male bottlenose dolphins (Tursiops sp.). Masters Thesis Univ Zurich

Bizzozzero MR, Allen SJ, Gerber L, et al (2019) Tool use and social homophily among male bottlenose dolphins. Proc R Soc B Biol Sci 286:20190898. https://doi.org/10.1098/rspb.2019.0898

Gilson A, Syvanen M, Levine K, Banks J (1998) Deer gender determination by polymerase chain reaction: Validation study and application to tissues, bloodstains, and hair forensic samples from California. Calif Fish Game

Krützen M, Barre LM, Moller LM, et al (2002) A biopsy system for small cetaceans: Darting success and wound healing in Tursiops spp. Mar Mammal Sci 18:863–878. https://doi.org/10.1111/j.1748-7692.2002.tb01078.x

Okonechnikov K, Golosova O, Fursov M, et al (2012) Unipro UGENE: A unified bioinformatics toolkit. Bioinformatics 28:1166–1167. https://doi.org/10.1093/bioinformatics/bts091

Oksanen J, Blanchet FG, Friendly M, et al (2018) Vegan: Community Ecology Package.

Smolker RA, Richards AF, Connor RC, Pepper JW (1992) Sex-differences in patterns of association among Indian-Ocean bottle-nosed dolphins. Behaviour 123:38–69. https://doi.org/10.1163/156853992x00101

Wang J (2011) Coancestry: A program for simulating, estimating and analysing relatedness and inbreeding coefficients. Mol Ecol Resour 11:141–145. https://doi.org/10.1111/j.1755-0998.2010.02885.x
