# Supplementary material for: Association patterns and community structure among female bottlenose dolphins: environmental, genetic and cultural factors
Source: Mamm Biol. 2022 Nov 2;102(4):1373–87. doi: 10.1007/s42991-022-00259-x (PMC10040398; doi:10.1007/s42991-022-00259-x)
Supplement: Supplementary file 5 — Supplementary file5 (PDF 209 KB) [file 42991_2022_259_MOESM5_ESM.pdf]

**Supplementary Material no. 5**

# Association patterns and community structure among female bottlenose dolphins: environmental, genetic and cultural factors

Svenja M. Marfurt*, Simon J. Allen, Manuela R. Bizzozzero, Erik P. Willems, Stephanie L. King, Richard C. Connor, Anna M. Kopps, Sonja Wild, Livia Gerber, Samuel Wittwer, Michael Krützen

* svenja.marfurt@uzh.ch

## Highlights:

- Extrinsic factors in combination with intrinsic behaviours appear to shape association patterns and community structure in female Indo-Pacific bottlenose dolphins (*Tursiops aduncus*)
- Dyadic associations are influenced by a combination of uni- and biparental relatedness, cultural behavior and habitat similarity (approximated by water depth)
- Members of the same communities overwhelmingly shared the same habitat and foraging techniques, demonstrating a strong homophilic tendency
- Intraspecific variation in sociality in bottlenose dolphins is thus influenced by a complex combination of genetic, cultural, and environmental aspects

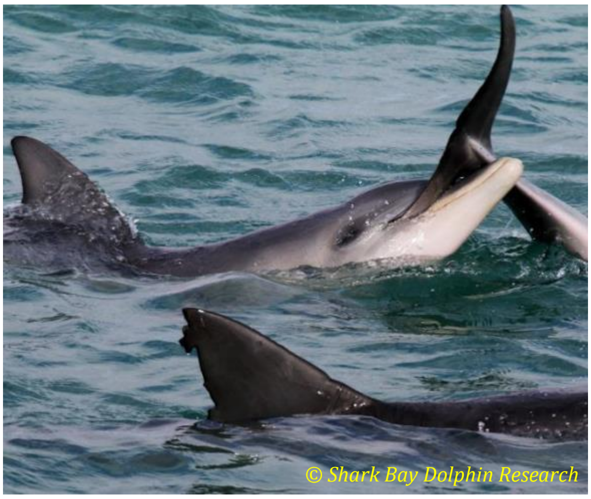


This article is part of a thematic collection of articles (Special Issue) of *Mammalian Biology* and covers the following topics and taxa (marked with ☑) addressed in the Special Issue:

| Article Type | | | | |
|---|---|---|---|---|
| ☑ Original Research | ☐ Techniques | ☐ Review | ☐ Short Communication | ☐ Concept Note |

| Taxon | | Topic | |
|---|---|---|---|
| ***Terrestrial*** | | | |
| ☐ Bats *(Order Chiroptera)* | ☐ Primates : Great Apes *(Family Hominidae)* | ☐ Acoustic ID | ☐ Identification techniques |
| ☐ Carnivores : Bears *(Family Ursidae)* | ☐ Primates : Old World monkeys *(Family Cercopithecidae)* | ☐ Aerial surveys | ☐ Life-history |
| ☐ Carnivores : Canids *(Family Canidae)* | ☐ Ungulates : Bovids *(Family Bovidae)* | ☐ Analytical innovations | ☐ Machine learning |
| ☐ Carnivores : Felids *(Family Felidae)* | ☐ Ungulates : Deers *(Family Cervidae)* | ☐ Automated pattern recognition | ☐ Mark-recapture analysis |
| ☐ Carnivores : Hyenas *(Family Hyaenidae)* | ☐ Ungulates : Giraffes *(Family Giraffidae)* | ☑ Behavioural ecology | ☐ Morphometrics |
| ☐ Carnivores : Mustelids *(Family Mustelidae)* | ☐ Ungulates : Horses *(Family Equidae)* | ☐ Camera-trapping | ☑ Network analysis |
| ☐ Elephants *(Family Elephantidae)* | ☐ Multiple taxa *(3 or more Families/Orders)* | ☐ Conservation management | ☐ Photogrammetry |
| ***Marine*** | | ☐ Data management | ☐ Population ecology |
| ☐ Baleen whales : Right whales *(Family Balaenidae)* | ☐ Large toothed whales *(Families Delphinidae & Hyperoodontidae)* | ☐ Demographic parameters | ☐ Site fidelity & Movement |
| ☐ Baleen whales : Rorquals *(Family Balaenopteridae)* | ☐ Pinnipeds : True seals *(Family Phocidae)* | ☐ Field methodology | ☑ Social ecology |
| ☐ Carnivores : Bears *(Family Ursidae)* | ☐ Porpoises *(Family Phocoenidae)* | ☐ Genetic ID | ☐ Software/Package development |
| ☐ Carnivores : Mustelids *(Family Mustelidae)* | ☐ Sirenians : Manatees *(Family Trichechidae)* | ☐ Health conditions | ☐ Thermal imagery |
| ☑ Dolphins *(Family Delphinidae)* | ☐ Multiple taxa *(3 or more Families/Orders)* | ☑ Other: Socio-genetics | |

## References

Karczmarski L, Chan SCY, Rubenstein DI, Chui SYS, Cameron EZ (2022a). Individual identification and photographic techniques in mammalian ecological and behavioural research – Part 1: Methods and concepts. *Mammalian Biology* (Special Issue), 102 (3) https://link.springer.com/journal/42991/volumes-and-issues/102-3

Karczmarski L, Chan SCY, Chui SYS, Cameron EZ (2022b). Individual identification and photographic techniques in mammalian ecological and behavioural research – Part 2: Field studies and applications. *Mammalian Biology* (Special Issue), 102 (4) https://link.springer.com/journal/42991/volumes-and-issues/102-4
